# Bis[2-(1*H*-imidazol-2-yl-κ*N* ^3^)-1*H*-imidazol-3-ium]silver(I) trinitrate

**DOI:** 10.1107/S1600536811021799

**Published:** 2011-06-11

**Authors:** Shelonda R. Finch, Johnathan P. Harper, Amitava Choudhury, Ekkehard Sinn, Harvest L. Collier

**Affiliations:** a Chemistry Department, Missouri University of Science and Technology, Rolla MO 65409, USA; b Western Michigan University, Chemistry Department, 1903 West Michigan Avenue MS 5413, Kalamazoo MI 49008, USA

## Abstract

The synthesis of the title salt, [Ag(C_6_H_7_N_4_)_2_](NO_3_)_3_, was carried out employing a 1:2 molar ratio of 2,2′-biimidazole and silver nitrate respectively. The cation has crystallographically-imposed *C*2 symmetry with the metal atom in an almost linear coordination environment [N—Ag—N = 177.01 (17)°]. The crystal structure displays N—H⋯O and C—H⋯O hydrogen-bonding inter­actions.

## Related literature

The synthesis of the complex is described by Hester *et al.* (1997 ▶). 2,2′-Biimidazole was prepared in a manner similar to Debus (1858 ▶).
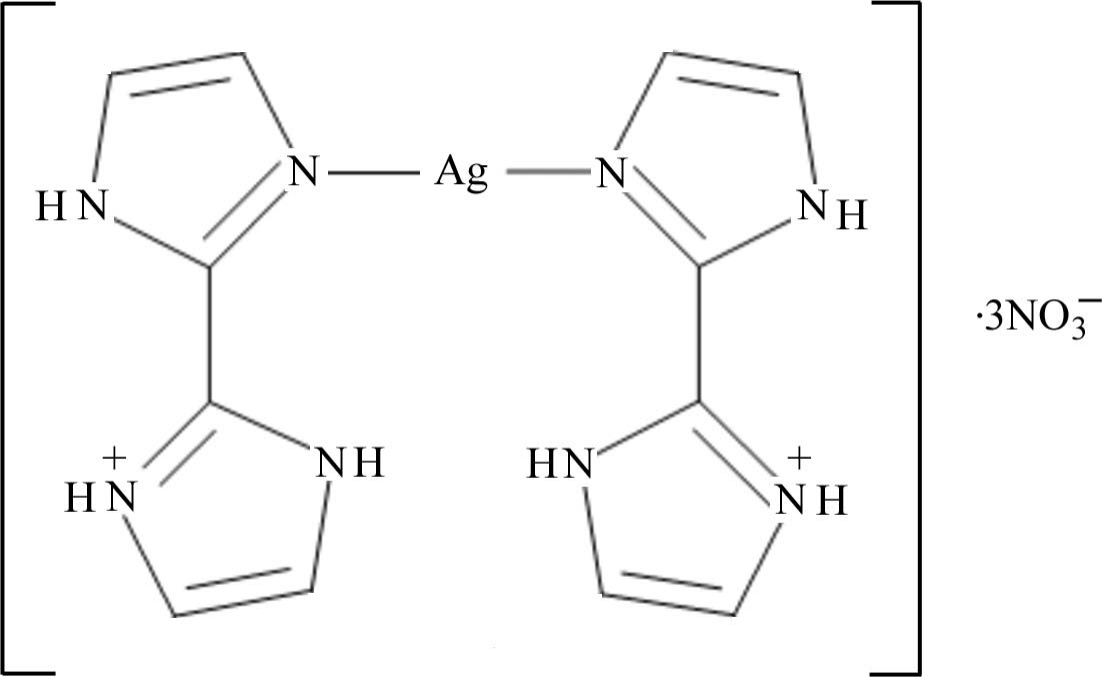


## Experimental

### 

#### Crystal data

[Ag(C_6_H_7_N_4_)_2_](NO_3_)_3_
*M* *_r_* = 564.21
Monoclinic, 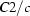

*a* = 24.095 (6) Å
*b* = 12.037 (3) Å
*c* = 6.8262 (18) Å
β = 91.319 (6)°
*V* = 1979.3 (9) Å^3^
*Z* = 4
Mo *K*α radiation
μ = 1.09 mm^−1^
*T* = 298 K
0.30 × 0.20 × 0.20 mm

#### Data collection

Bruker SMART APEX CCD area-detector diffractometer
Absorption correction: multi-scan (*SADABS*; Bruker, 2008 ▶) *T*_min_ = 0.735, *T*_max_ = 0.811
9412 measured reflections
2275 independent reflections
1723 reflections with *I* > 2σ(*I*)
*R*_int_ = 0.065

#### Refinement

*R*[*F* ^2^ > 2σ(*F* ^2^)] = 0.044
*wR*(*F* ^2^) = 0.104
*S* = 0.99
2275 reflections
151 parameters
H-atom parameters constrained
Δρ_max_ = 0.46 e Å^−3^
Δρ_min_ = −0.34 e Å^−3^

### 

Data collection: *SMART* (Bruker, 2002 ▶); cell refinement: *SAINT* (Bruker, 2008 ▶); data reduction: *SAINT*; program(s) used to solve structure: *SHELXS97* (Sheldrick, 2008 ▶); program(s) used to refine structure: *SHELXL97* (Sheldrick, 2008 ▶); molecular graphics: *SHELXTL* (Sheldrick, 2008 ▶); software used to prepare material for publication: *SHELXTL*.

## Supplementary Material

Crystal structure: contains datablock(s) I, global. DOI: 10.1107/S1600536811021799/mw2006sup1.cif

Structure factors: contains datablock(s) I. DOI: 10.1107/S1600536811021799/mw2006Isup2.hkl

Additional supplementary materials:  crystallographic information; 3D view; checkCIF report

## Figures and Tables

**Table 1:** Hydrogen-bond geometry (Å, °)

*D*—H⋯*A*	*D*—H	H⋯*A*	*D*⋯*A*	*D*—H⋯*A*
N2—H2*N*⋯O4^i^	0.86	1.94	2.792 (4)	173
N3—H3⋯O1^ii^	0.86	1.92	2.765 (4)	166
N4—H4⋯O4	0.86	1.93	2.758 (4)	160
C1—H1⋯O3^ii^	0.93	2.49	3.179 (5)	131
C2—H2⋯O5^iii^	0.93	2.60	3.331 (5)	136
C5—H5⋯O3^iv^	0.93	2.55	3.340 (6)	144
C6—H6⋯O2^v^	0.93	2.55	3.376 (6)	148

Symmetry codes: (i) 
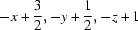
; (ii) 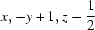; (iii) 
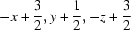
; (iv) 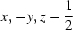; (v) 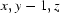.

**Fig. 1.**
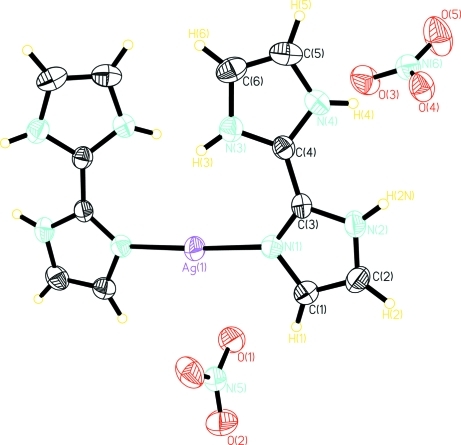
Asymmetric unit of silver bis(1H-imidazolium) trinitrate. Thermal ellipsoids are drawn at the 40% probability level.

**Fig. 2.**
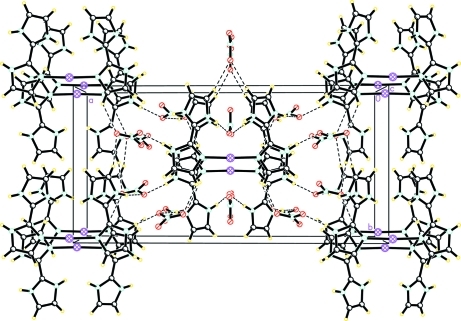
Unit cell packing of silver bis(1H-imidazolium) trinitrate shown along the c-axis. Dotted lines indicate N—H···O and C—H···O hydrogen-bonding interactions.

## supplementary crystallographic information

### Comment

The compound, silver bis (1*H*-imidazolium) trinitrate [Ag(biimH)_2_(NO_3_)_3_] crystallizes in the monoclinic crystal system in space group *C2/c*. The Ag atom is coordinated to N atoms on two different 2,2'-biimidazole entities in an almost linear geometry where the biimidazole acts as a monodentate ligand. The Ag—N distance is 2.118 (3) Å and the N—Ag—N bond angle is 177.01 (17)°. There are one and one half crystallographically independent nitrate anions in the asymmetric unit (Figure 1). The nearest contacts of Ag with O atoms from one of the nitrate groups are in the range 3.026 (3)–3.070 (3) Å. The other nitrate is hydrogen-bonded to the other side of the uncoordinated ring of 2,2'-bimidazole (Figure 2). The two imidazole rings in 2,2'-biimidazole are no longer in one plane when coordinated to Ag as in [Ag(biimH)_2_(NO_3_)_3_] but are twisted with a N1—C3—C4—N4 torsion angle of 33.5 (6)°. A very similar torsion angle [34.30 (7)°] has been observed in another silver complex of 2,2'-biimidazole (Hester *et al.* (1997)) where such a torsion angle between the imidazole rings is responsible for the helicity of the complex. However, in contrast to the present structure, in the Ag-biimidazole complex reported by Hester the biimidazole binds to Ag atoms in a bis-monodentate fashion bridging two Ag atoms with a very close Ag—Ag contact distance of 3.003 (3)Å and an N—Ag—N bond angle of 162.36 (2)°. This contrasts with the nearest Ag—Ag contact distance in the present complex which is considerably greater at 3.5473 (9) Å.

### Experimental

2,2'-Biimidazole was prepared in a manner similar to Debus (1858), using equal portions of 40% glyoxal and concentrated ammonium hydroxide (28–30%). Silver nitrate was used as received and concentrated nitric acid was diluted to 0.1 *M*. The synthesis of silver bis(1*H*-imidazolium) trinitrate used a procedure similar to that reported by Hester *et al.* (1997), using 0.1 *M* HNO_3_. A mass of 1.343 g (1.001 *x* 10 ^-2^ mol) of 2,2'-biimidazole was dissolved in 15 ml of 0.1*M* HNO_3_ using heat. Silver nitrate (3.410 g, 2.007 *x* 10 ^-2^ mol) was then added to the solution as a solid. A precipitate formed upon mixing and a few drops of 0.1 *M* HNO_3_ were added to resolubilize the precipitate. Colourless crystals formed as the solution was allowed to slowly evaporate.

### Refinement

Hydrogen atoms were placed geometrically and held in the riding mode during the final refinement. C—H = 0.93 Å with *U*iso (H) = 1.2*U*eq(C) and N—H = 0.86 Å with *U*iso (H) = 1.2*U*eq(N).

### Crystal data

**Table:**

[Ag(C_6_H_7_N_4_)_2_](NO_3_)_3_	*F*(000) = 1128
*M**_r_* = 564.21	*D*_x_ = 1.893 Mg m^−^^3^
Monoclinic, *C*2/*c*	Mo *K*α radiation, λ = 0.71073 Å
Hall symbol: -C 2yc	Cell parameters from 2422 reflections
*a* = 24.095 (6) Å	θ = 3.1–24.0°
*b* = 12.037 (3) Å	µ = 1.09 mm^−^^1^
*c* = 6.8262 (18) Å	*T* = 298 K
β = 91.319 (6)°	Needle, colourless
*V* = 1979.3 (9) Å^3^	0.30 × 0.20 × 0.20 mm
*Z* = 4	

### Data collection

**Table:**

Bruker SMART APEX CCD area-detector diffractometer	2275 independent reflections
Radiation source: fine-focus sealed tube	1723 reflections with *I* > 2σ(*I*)
graphite	*R*_int_ = 0.065
ω scans	θ_max_ = 27.5°, θ_min_ = 1.7°
Absorption correction: multi-scan (*SADABS*; Bruker, 2008)	*h* = −31→31
*T*_min_ = 0.735, *T*_max_ = 0.811	*k* = −15→15
9412 measured reflections	*l* = −8→8

### Refinement

**Table:**

Refinement on *F*^2^	Primary atom site location: structure-invariant direct methods
Least-squares matrix: full	Secondary atom site location: difference Fourier map
*R*[*F*^2^ > 2σ(*F*^2^)] = 0.044	Hydrogen site location: inferred from neighbouring sites
*wR*(*F*^2^) = 0.104	H-atom parameters constrained
*S* = 0.99	*w* = 1/[σ^2^(*F*_o_^2^) + (0.0505*P*)^2^] where *P* = (*F*_o_^2^ + 2*F*_c_^2^)/3
2275 reflections	(Δ/σ)_max_ < 0.001
151 parameters	Δρ_max_ = 0.46 e Å^−^^3^
0 restraints	Δρ_min_ = −0.34 e Å^−^^3^

### Special details

**Table:**

Geometry. All e.s.d.'s (except the e.s.d. in the dihedral angle between two l.s. planes) are estimated using the full covariance matrix. The cell e.s.d.'s are taken into account individually in the estimation of e.s.d.'s in distances, angles and torsion angles; correlations between e.s.d.'s in cell parameters are only used when they are defined by crystal symmetry. An approximate (isotropic) treatment of cell e.s.d.'s is used for estimating e.s.d.'s involving l.s. planes.
Refinement. Refinement of *F*^2^ against ALL reflections. The weighted *R*-factor *wR* and goodness of fit *S* are based on *F*^2^, conventional *R*-factors *R* are based on *F*, with *F* set to zero for negative *F*^2^. The threshold expression of *F*^2^ > σ(*F*^2^) is used only for calculating *R*-factors(gt) *etc*. and is not relevant to the choice of reflections for refinement. *R*-factors based on *F*^2^ are statistically about twice as large as those based on *F*, and *R*- factors based on ALL data will be even larger.

### Fractional atomic coordinates and isotropic or equivalent isotropic displacement parameters (Å^2^)

**Table:**

	*x*	*y*	*z*	*U*_iso_*/*U*_eq_	
Ag1	0.5000	0.45986 (4)	0.2500	0.04700 (17)	
N1	0.58789 (12)	0.4553 (3)	0.2547 (4)	0.0405 (7)	
C1	0.61871 (16)	0.5520 (3)	0.2559 (6)	0.0459 (9)	
H1	0.6044	0.6235	0.2442	0.055*	
C2	0.67332 (17)	0.5259 (3)	0.2768 (6)	0.0510 (10)	
H2	0.7029	0.5755	0.2832	0.061*	
N2	0.67642 (13)	0.4139 (3)	0.2867 (5)	0.0468 (8)	
H2N	0.7063	0.3753	0.2994	0.056*	
C3	0.62405 (14)	0.3734 (3)	0.2730 (5)	0.0379 (8)	
C4	0.61183 (14)	0.2557 (3)	0.2817 (5)	0.0378 (8)	
N3	0.57064 (13)	0.2026 (3)	0.1904 (5)	0.0440 (8)	
H3	0.5456	0.2331	0.1165	0.053*	
C6	0.57421 (18)	0.0915 (3)	0.2320 (6)	0.0527 (10)	
H6	0.5506	0.0358	0.1856	0.063*	
C5	0.6184 (2)	0.0791 (4)	0.3527 (7)	0.0590 (12)	
H5	0.6310	0.0126	0.4068	0.071*	
N4	0.64157 (14)	0.1813 (3)	0.3822 (5)	0.0487 (8)	
H4	0.6706	0.1952	0.4538	0.058*	
O1	0.50331 (14)	0.6989 (3)	0.4067 (5)	0.0721 (10)	
N5	0.5000	0.7527 (5)	0.2500	0.0511 (12)	
O2	0.5000	0.8540 (4)	0.2500	0.0879 (16)	
O3	0.64511 (13)	0.1930 (3)	0.8363 (5)	0.0738 (10)	
N6	0.69447 (15)	0.1706 (3)	0.8314 (6)	0.0541 (9)	
O4	0.72151 (11)	0.1948 (3)	0.6770 (4)	0.0579 (8)	
O5	0.71807 (15)	0.1225 (4)	0.9651 (5)	0.0968 (13)	

### Atomic displacement parameters (Å^2^)

**Table:**

	*U*^11^	*U*^22^	*U*^33^	*U*^12^	*U*^13^	*U*^23^
Ag1	0.0336 (2)	0.0475 (3)	0.0597 (3)	0.000	−0.00185 (17)	0.000
N1	0.0385 (16)	0.0417 (17)	0.0412 (17)	−0.0022 (14)	−0.0010 (13)	0.0016 (14)
C1	0.047 (2)	0.042 (2)	0.049 (2)	−0.0031 (17)	0.0005 (17)	−0.0007 (18)
C2	0.048 (2)	0.053 (3)	0.052 (2)	−0.0141 (19)	−0.0009 (18)	0.002 (2)
N2	0.0330 (16)	0.056 (2)	0.051 (2)	−0.0003 (14)	−0.0034 (14)	0.0038 (16)
C3	0.0326 (17)	0.045 (2)	0.0365 (19)	0.0007 (15)	−0.0030 (14)	−0.0024 (16)
C4	0.0351 (18)	0.042 (2)	0.036 (2)	0.0031 (15)	0.0022 (15)	0.0011 (16)
N3	0.0427 (18)	0.0475 (19)	0.0416 (18)	−0.0023 (14)	−0.0009 (14)	−0.0004 (14)
C6	0.061 (3)	0.042 (2)	0.056 (3)	−0.006 (2)	0.016 (2)	−0.007 (2)
C5	0.071 (3)	0.041 (2)	0.066 (3)	0.008 (2)	0.012 (2)	0.007 (2)
N4	0.0499 (19)	0.049 (2)	0.047 (2)	0.0066 (15)	−0.0035 (15)	0.0055 (16)
O1	0.090 (2)	0.073 (2)	0.0526 (19)	−0.0163 (19)	−0.0198 (17)	0.0118 (16)
N5	0.047 (3)	0.052 (3)	0.054 (3)	0.000	−0.005 (2)	0.000
O2	0.095 (4)	0.046 (3)	0.122 (5)	0.000	−0.009 (3)	0.000
O3	0.053 (2)	0.078 (2)	0.091 (3)	0.0165 (17)	0.0225 (17)	0.0085 (19)
N6	0.049 (2)	0.054 (2)	0.059 (2)	0.0017 (17)	0.0028 (18)	0.0014 (18)
O4	0.0448 (16)	0.076 (2)	0.0532 (18)	0.0034 (14)	0.0059 (13)	0.0107 (15)
O5	0.074 (2)	0.149 (4)	0.067 (2)	0.007 (3)	−0.0109 (19)	0.039 (2)

### Geometric parameters (Å, °)

**Table:**

Ag1—N1^i^	2.118 (3)	N3—C6	1.370 (5)
Ag1—N1	2.118 (3)	N3—H3	0.8600
N1—C3	1.319 (4)	C6—C5	1.340 (6)
N1—C1	1.381 (5)	C6—H6	0.9300
C1—C2	1.357 (6)	C5—N4	1.365 (5)
C1—H1	0.9300	C5—H5	0.9300
C2—N2	1.351 (5)	N4—H4	0.8600
C2—H2	0.9300	O1—N5	1.251 (4)
N2—C3	1.354 (5)	N5—O2	1.220 (7)
N2—H2N	0.8600	N5—O1^i^	1.251 (4)
C3—C4	1.449 (5)	O3—N6	1.221 (4)
C4—N3	1.324 (5)	N6—O5	1.211 (5)
C4—N4	1.328 (5)	N6—O4	1.285 (4)
			
N1^i^—Ag1—N1	177.01 (17)	C4—N3—C6	109.3 (3)
C3—N1—C1	106.0 (3)	C4—N3—H3	125.3
C3—N1—Ag1	132.8 (2)	C6—N3—H3	125.3
C1—N1—Ag1	121.0 (2)	C5—C6—N3	106.4 (4)
C2—C1—N1	109.0 (4)	C5—C6—H6	126.8
C2—C1—H1	125.5	N3—C6—H6	126.8
N1—C1—H1	125.5	C6—C5—N4	107.8 (4)
N2—C2—C1	106.8 (4)	C6—C5—H5	126.1
N2—C2—H2	126.6	N4—C5—H5	126.1
C1—C2—H2	126.6	C4—N4—C5	108.6 (3)
C2—N2—C3	107.8 (3)	C4—N4—H4	125.7
C2—N2—H2N	126.1	C5—N4—H4	125.7
C3—N2—H2N	126.1	O2—N5—O1^i^	121.2 (3)
N1—C3—N2	110.5 (3)	O2—N5—O1	121.2 (3)
N1—C3—C4	126.9 (3)	O1^i^—N5—O1	117.6 (5)
N2—C3—C4	122.6 (3)	O5—N6—O3	121.7 (4)
N3—C4—N4	107.9 (3)	O5—N6—O4	119.2 (4)
N3—C4—C3	127.1 (3)	O3—N6—O4	119.0 (4)
N4—C4—C3	125.0 (3)		
			
C3—N1—C1—C2	−0.5 (4)	N2—C3—C4—N3	−147.8 (4)
Ag1—N1—C1—C2	174.5 (3)	N1—C3—C4—N4	−148.0 (4)
N1—C1—C2—N2	0.6 (5)	N2—C3—C4—N4	30.8 (6)
C1—C2—N2—C3	−0.4 (4)	N4—C4—N3—C6	−0.4 (4)
C1—N1—C3—N2	0.3 (4)	C3—C4—N3—C6	178.3 (4)
Ag1—N1—C3—N2	−173.9 (2)	C4—N3—C6—C5	0.6 (5)
C1—N1—C3—C4	179.2 (4)	N3—C6—C5—N4	−0.6 (5)
Ag1—N1—C3—C4	5.0 (6)	N3—C4—N4—C5	0.0 (4)
C2—N2—C3—N1	0.0 (4)	C3—C4—N4—C5	−178.7 (4)
C2—N2—C3—C4	−178.9 (3)	C6—C5—N4—C4	0.3 (5)
N1—C3—C4—N3	33.5 (6)		

Symmetry codes: (i) −*x*+1, *y*, −*z*+1/2.

### Hydrogen-bond geometry (Å, °)

**Table:**

*D*—H···*A*	*D*—H	H···*A*	*D*···*A*	*D*—H···*A*
N2—H2N···O4^ii^	0.86	1.94	2.792 (4)	173
N3—H3···O1^iii^	0.86	1.92	2.765 (4)	166
N4—H4···O4	0.86	1.93	2.758 (4)	160
C1—H1···O3^iii^	0.93	2.49	3.179 (5)	131
C2—H2···O5^iv^	0.93	2.60	3.331 (5)	136
C5—H5···O3^v^	0.93	2.55	3.340 (6)	144
C6—H6···O2^vi^	0.93	2.55	3.376 (6)	148

Symmetry codes: (ii) −*x*+3/2, −*y*+1/2, −*z*+1; (iii) *x*, −*y*+1, *z*−1/2; (iv) −*x*+3/2, *y*+1/2, −*z*+3/2; (v) *x*, −*y*, *z*−1/2; (vi) *x*, *y*−1, *z*.
